# A three-dimensional atlas of child’s cardiac anatomy and the unique morphological alterations associated with obesity

**DOI:** 10.1093/ehjci/jeab271

**Published:** 2021-12-21

**Authors:** Maciej Marciniak, Arend W van Deutekom, Liza Toemen, Adam J Lewandowski, Romy Gaillard, Alistair A Young, Vincent W V Jaddoe, Pablo Lamata

**Affiliations:** Department of Biomedical Engineering, School of Biomedical Engineering and Imaging Sciences, Kings’ College London, 5th Floor Becket House, Lambeth Palace Road, London SE1 7EU, UK; Department of Paediatrics, Erasmus MC, University Medical Center, Rotterdam, the Netherlands; Department of Paediatrics, Generation R Study Group, Erasmus MC, University Medical Center, Wytemaweg 80, 3015 CN Rotterdam, the Netherlands; Division of Cardiovascular Medicine, Radcliffe Department of Medicine, Oxford Cardiovascular Clinical Research Facility, University of Oxford, Level 1 Oxford Heart Centre, John Radliffe Hospital, Headley Way, Headington, Oxford, OX3 9DU, UK; Department of Paediatrics, Erasmus MC, University Medical Center, Rotterdam, the Netherlands; Department of Paediatrics, Generation R Study Group, Erasmus MC, University Medical Center, Wytemaweg 80, 3015 CN Rotterdam, the Netherlands; Department of Epidemiology, Erasmus MC, University Medical Center, Dr. Molewaterplein 40, 3015 GD Rotterdam, the Netherlands; Division of Cardiovascular Medicine, Radcliffe Department of Medicine, Oxford Cardiovascular Clinical Research Facility, University of Oxford, Level 1 Oxford Heart Centre, John Radliffe Hospital, Headley Way, Headington, Oxford, OX3 9DU, UK; Department of Paediatrics, Erasmus MC, University Medical Center, Rotterdam, the Netherlands; Department of Paediatrics, Generation R Study Group, Erasmus MC, University Medical Center, Wytemaweg 80, 3015 CN Rotterdam, the Netherlands; Department of Biomedical Engineering, School of Biomedical Engineering and Imaging Sciences, Kings’ College London, 5th Floor Becket House, Lambeth Palace Road, London SE1 7EU, UK; Department of Paediatrics, Erasmus MC, University Medical Center, Rotterdam, the Netherlands; Department of Paediatrics, Generation R Study Group, Erasmus MC, University Medical Center, Wytemaweg 80, 3015 CN Rotterdam, the Netherlands; Department of Epidemiology, Erasmus MC, University Medical Center, Dr. Molewaterplein 40, 3015 GD Rotterdam, the Netherlands; Department of Biomedical Engineering, School of Biomedical Engineering and Imaging Sciences, Kings’ College London, 5th Floor Becket House, Lambeth Palace Road, London SE1 7EU, UK

**Keywords:** paediatrics, obesity, risk factors, magnetic resonance imaging, epidemiology

## Abstract

**Aims:**

Statistical shape models (SSMs) of cardiac anatomy provide a new approach for analysis of cardiac anatomy. In adults, specific cardiac morphologies associate with cardiovascular risk factors and early disease stages. However, the relationships between morphology and risk factors in children remain unknown. We propose an SSM of the paediatric left ventricle to describe its morphological variability, examine its relationship with biometric parameters and identify adverse anatomical remodelling associated with obesity.

**Methods and results:**

This cohort includes 2631 children (age 10.2 ± 0.6 years), mostly Western European (68.3%) with a balanced sex distribution (51.3% girls) from Generation R study. Cardiac magnetic resonance short-axis cine scans were segmented. Three-dimensional left ventricular (LV) meshes are automatically fitted to the segmentations to reconstruct the anatomies. We analyse the relationships between the LV anatomical features and participants’ body surface area (BSA), age, and sex, and search for features uniquely related to obesity based on body mass index (BMI). In the SSM, 19 modes described over 90% of the population’s LV shape variability. Main modes of variation were related to cardiac size, sphericity, and apical tilting. BSA, age, and sex were mostly correlated with modes describing LV size and sphericity. The modes correlated uniquely with BMI suggested that obese children present with septo-lateral tilting (*R*^2^ = 4.0%), compression in the antero-posterior direction (*R*^2^ = 3.3%), and decreased eccentricity (*R*^2^ = 2.0%).

**Conclusions:**

We describe the variability of the paediatric heart morphology and identify anatomical features related to childhood obesity that could aid in risk stratification. Web service is released to provide access to the new shape parameters.

## Graphical Abstract

**Graphical Abstract:**
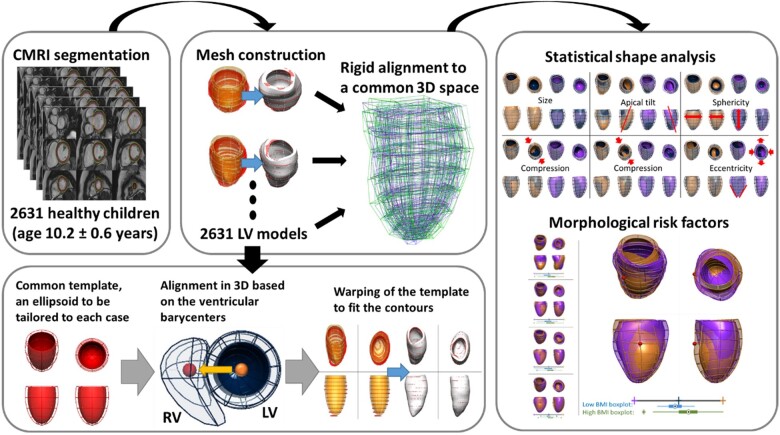

## Introduction

Obesity remains one of the most important issues in global health. Almost 60% of EU adults and roughly one-third of 11-year-olds are overweight or obese and obesity-related conditions are the leading causes of preventable death.^1,2^ The increased cardiovascular disease (CVD) risk associated with obesity is not only driven by hypertension, diabetes, obstructive sleep apnoea, and coronary artery disease but also by structural and functional cardiac changes.^3^

Three-dimensional (3D) statistical shape models (SSMs) of cardiac anatomy using cardiovascular magnetic resonance (CMR) images offer a new approach for accurate quantitative assessment of cardiac geometry, suitable for large cohort studies.^4^ For example, SSM have been used to unveil changes in left ventricular (LV) geometry in healthy adults who were born prematurely,^5^ to quantify remodelling patterns after myocardial infarction,^6^ to predict response after cardiac resynchronization therapy,^7^ and to detect early signs of heart failure in congenital heart disease.^8^

The application of SSM in large cohorts found that obese adults have a distinct cardiac shape that may better explain the interaction between body composition and cardiac health. In the UK Digital Heart Project obesity was associated with asymmetric concentric hypertrophy, with women demonstrating greater cavity dilatation than men.^9^ In the UK Biobank study, shape variants characterized by septal displacement and apical bulging had robust and strong relationships with obesity.^10^ In both cohorts, these new measures of cardiac shape were more sensitive to detect cardiac differences associated with body mass index (BMI) than traditional measures such as LV mass (LVM) and LV end-diastolic volume (LVEDV) and were independently associated with risk factors for later CVD.

Part of the adult CVD risk associated with obesity may already develop in childhood. The Bogalusa Heart Study revealed that childhood obesity is associates with adult LV dilatation and hypertrophy and concluded that childhood BMI was the *only* independent predictor of LV hypertrophy in adulthood.^11^ Epidemiological studies in children indicate that LV hypertrophy develops in response to obesity in childhood,^12,13^ and relates to an increase in myocardial triglyceride content in adolescence.^14^

In this study, we describe the construction of an SSM from >2600 children of the Generation R study to provide a reference standard of the paediatric LV shape. We define how different 3D shape features relate to conventional geometric measurements and biometric parameters. We hypothesize that the shape analysis can reveal an anatomical LV remodelling pattern that is uniquely associated with paediatric obesity. This knowledge may have implications for screening, prevention, and understanding the increased cardiovascular risk in obesity.

## Methods

This study draws from the Generation R study, a population-based prospective cohort study from foetal life onwards in the Netherlands.^15^ Extensive data collection has been conducted over the years, from the early prenatal phase through childhood, to examine the development of the cardiovascular system and early cardiovascular risk factors.^15,16^ Data collection in parents and their children included questionnaires, physical and ultrasound examinations, and CMR imaging scans. Of the initial cohort of 9749 live births, 3719 children participated in the CMR studies around the age of 10 years [min = 8.55, max = 11.99, median = 9.95, interquartile range (9.77, 10.35)]. Children’s height and weight was measured (see Supplementary data online) to derive body surface area (BSA), using the Haycock formula,^17^ sex- and age-specific BMI Z-scores (based on national reference diagrams^18^), and weight status (based on the definition of the International Obesity Task Force^19^).

For CMR imaging, we used a standardized protocol (see Supplementary data online) to obtain a short-axis steady state free precession cine stack covering the ventricles with 8-mm thick slices. LV endo- and epicardial borders were semi-automatically contoured using QMASS software (Medis, Leiden, the Netherlands). Traditional cardiac outcome parameters included LVEDV and LVM, based on the Simpson’s method.

### Computational models

**Figure 1:**
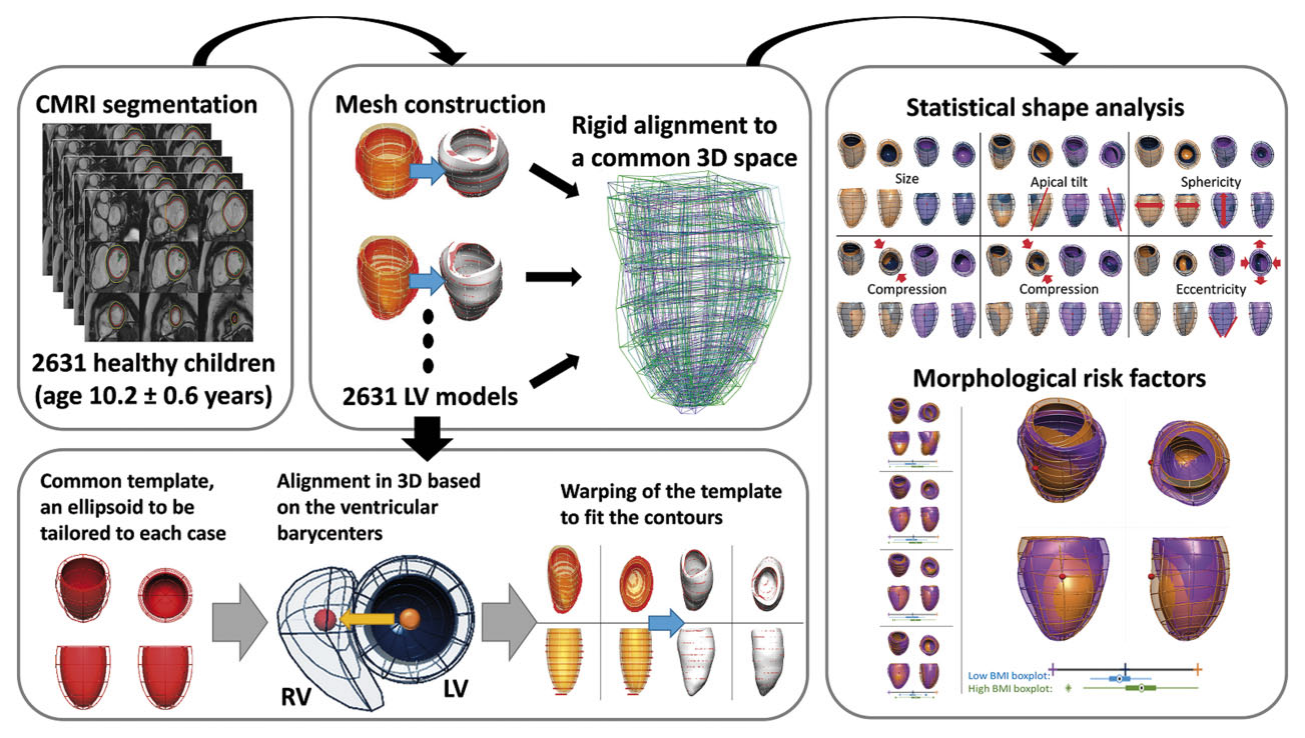
Building a statistical shape model for morphological variability description and risk factors detection. Cardiac magnetic resonance short-axis cine scans from 2631 children (age 10.2 ± 0.6 years, 51% girls) were segmented. 3D LV meshes were automatically fitted to the segmentations to reconstruct the LV anatomies and registered to ensure the anatomical correspondence of each mesh. Then, a statistical shape model was computed to derive modes of shape variation. Nineteen modes described over 90% of the LV shape variability within the population. Body surface area, age, and sex were mostly correlated with modes describing LV size and sphericity. Few modes were correlated uniquely with body mass index, suggesting that obese children present with septo-lateral tilting (*R*^2^ = 4.0%), compression in the antero-posterior direction (*R*^2^ = 3.3%), and decreased eccentricity (*R*^2^ = 2.0%).

The LV 3D anatomy of each subject was reconstructed from the contours of the left ventricle at end-diastole in the short-axis stack. An anatomical personalization method that uses smooth meshes was used due to its robustness to segmentation variability.^20,21^ In short, this method customizes an idealized LV template to the contours using image registration and mesh warping techniques and achieves sub-voxel accuracy. Anatomical correspondence across meshes was achieved by the consistent alignment between template and patient-specific contours at the initial step of the personalization process. This alignment was based on the direction perpendicular to the CMR short-axis basal plane and on the direction joining the centre of mass of the right ventricular and LV blood pools (see *Figure 1*).

Geometric measurements were performed on the generated meshes. LVEDV and LVM were computed from meshes by numerical integration and using the myocardial density of 1.055 g/mL.^22^ LV mass to volume ratio (MVR) was computed from these two (LVM/LVEDV). LV length was computed from the centre of the basal plane to the furthest apical epicardial point. LV diameter was calculated as the average of the diameters of circles inscribed into the epicardial cross-section at 40–50% of the LV length, measured from the base. LV sphericity was calculated as a ratio of the LV diameter and LV length. LV eccentricity was calculated as a ratio of the endocardial diameters parallel and perpendicular to the interventricular septum and passing through the centre of the cavity at 10% of the LV length measured from the base.^23^

The accuracy of the generated meshes was evaluated by the distance from their surfaces to the initial contours, and by comparison of their LVEDV and LVM with the corresponding traditional measurement.

### Statistical shape modelling

An SSM was built to study the variations of the anatomy with respect to their average as reported previously.^5^ In brief, since each anatomy was represented by a large set of degrees of freedom (3120 values encoding 130 points and derivatives in the 3D space), we reduced the dimensionality using principal component analysis (PCA). PCA finds the axes of anatomical change (modes) that maximize the amount of variance explained and that are not correlated with one another. PCA modes are sorted by the amount of variance they explain, and a reduced (about 100-fold) number of them is sufficient to encode for 3D shape variations observed. Two first modes were adjusted to improve interpretability (Supplementary data online, *Figure S1*).

The number of PCA modes to be included in this study was defined by two criteria: capturing at least 90% of the variability in shape and explaining at least 90% of the variability in conventional geometrical metrics (LVEDV, LVM, MVR, and length).

### Statistical analysis

Characteristics of the cohort were summarized as means and standard deviations (SDs) for continuous data and as frequency distributions for categorical data. Linear regression analysis was performed to discover the significant correlations between PCA modes and biometric parameters. PCA modes uniquely related to obesity were defined as being correlated with BMI scores (coefficient of determination *R*^2^ > 1.4%) and at least two times more correlated with BMI than with BSA ($R_{\mathrm{BMI}}^{2}>2*R_{\mathrm{BSA}}^{2}$). Linear discriminant analysis was used to find the anatomical remodelling pattern associated with obesity, by searching for the linear combination of PCA modes that best separate cases with overweight and obesity and cases with average BMI (mean ± 0.1*SD).

The statistical analysis and the variability analysis were implemented using the Python programming language, v.3.6.5. Two sample *t*-test is employed to test hypotheses. *P* < 0.001 in both regression analysis and hypothesis testing were considered significant. The data underlying this article will be shared upon reasonable request to the corresponding author.

## Results

### Cohort characteristics

We obtained acceptable quality CMR data, suitable for our 3D analysis in 2631 children (see Supplementary data online, *Figure S2*). Baseline characteristics of these children are shown in *Table 1*. A total of *n* = 185 individuals were overweight or obese and were more often girls and not of Western European ethnicity (see Supplementary data online, *Table S1*). These children also had a higher LVEDV and LVM.^13^

**Table 1:** Baseline characteristics of study participants

Variable	Average (SD)
Number of participants	2631
Age at MRI, years	10.2 (0.6)
Male sex, *N* (%)	1281 (48.7%)
Western European ethnicity, *N* (%)	1796 (68.3%)
Height, cm	141.7 (6.6)
Weight, kg	35.3 (7.1)
Body mass index, kg/m^2^	17.5 (2.7)
Underweight, *N* (%)	49 (1.9%)
Overweight or obese, *N* (%)	185 (7.0%)
Body surface area, m^2^	1.17 (0.14)
Left ventricular end-diastolic volume, mL	100.6 (17.5)
Left ventricular mass, g	48.8 (10.2)

MRI, magnetic resonance imaging; SD, standard deviation.

### Major modes of shape variation in the paediatric LV

**Figure 2:**
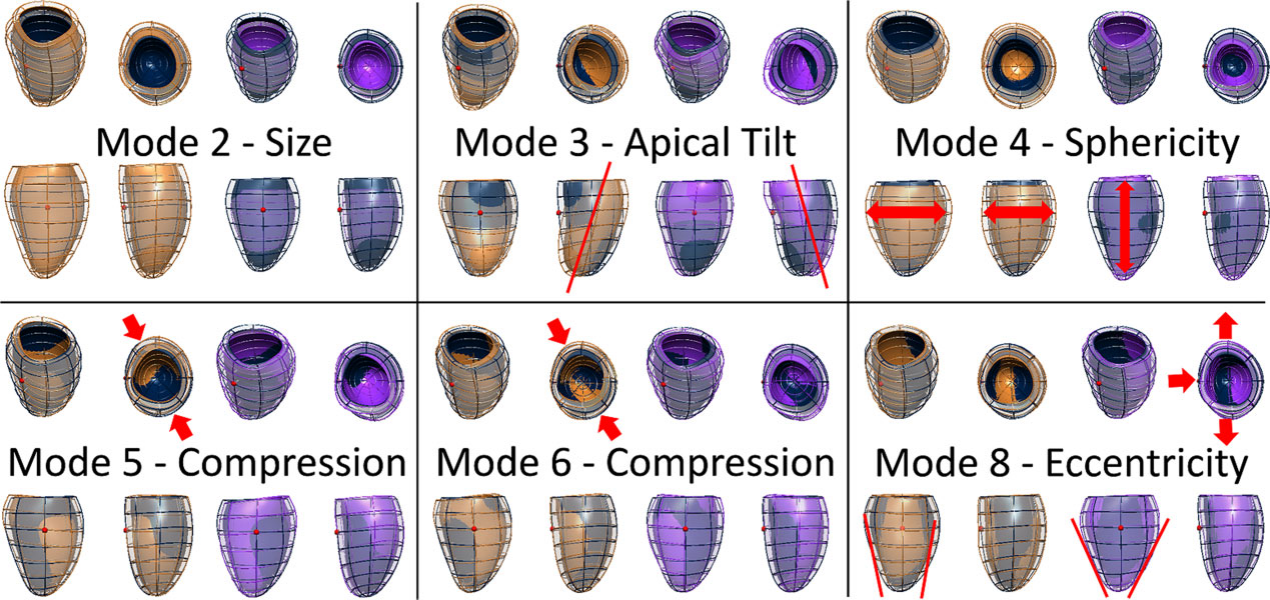
The most relevant shape modes generated with the statistical shape analysis. Dark blue—average mesh, orange, and purple—plus and minus 3 standard deviations from the average mesh along the shape mode. Modes 2–4 described the variation in size, orientation, and sphericity of the ventricle. Modes 5 and 6 related to antero/inferoseptal compression, and mode 8 associated with eccentricity of the LV.

The 3D LV meshes showed a good agreement in LVM and LVEDV resulting from the contours (see Supplementary data online, *Figure S3*). The first 19 PCA modes explained 90.6% of the variability in LV shape, and captured a total *R*^2^ of 99%, 95%, 91%, and 92% with LVEDV, LVM, MVR, and LV length, respectively. The shape modes that were the most relevant for this study are depicted in *Figure 2*. The first four modes were associated with size (mode 2), tilting in the apico-basal axis (modes 1 and 3, corresponding to antero-posterior and septo-lateral tilting respectively), and sphericity (mode 4). Modes 5 and 6 were associated with compression in the anteroposterior direction, and mode 8 manifested the change in eccentricity of the LV cross-section. Visualization of the first 19 modes is provided in Supplementary data online, *Tables S2* and *S3*, and the Supplementary data online, *ModeVideos.pptx*.

**Figure 3:**
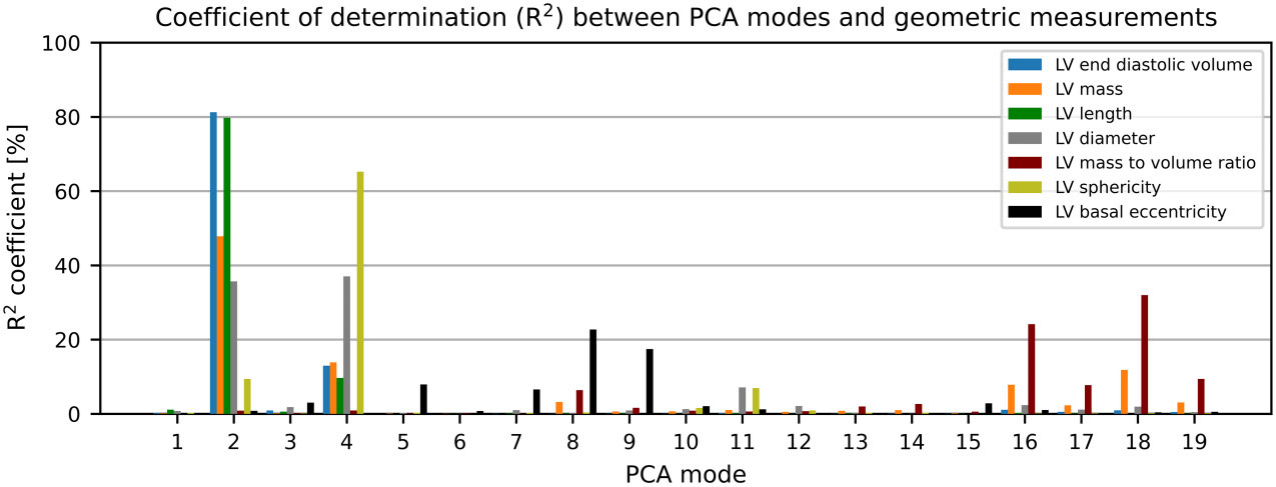
Correlation between PCA modes and LV geometric descriptors. LVEDV is explained by modes 2 and 4 up to a *R*^2^ of over 94%. Modes 2 and 4 are also the main determinants of LVM and sphericity. LVM and MVR depend on modes 16–19 that describe 1.94% of the overall shape variance. LVEDV, left ventricular end-diastolic volume; LVM, left ventricular mass; PCA, principal component analysis.

The correlations between the first 19 modes and conventional geometrical measures are shown in *Figure 3*. All the correlations described here and in the following subsections were statistically significant (*P* < 0.001). Modes 2 and 4 were the most relevant to capture changes in LVEDV, LVM, and sphericity without changing MVR. LVEDV was explained by these two modes with *R*^2^ = 94.2% [81.2%, 95% confidence interval: (79.9%, 82.5%) and 13.0% (10.6%, 15.4%) for modes 2 and 4, respectively], sphericity with *R*^2^ = 74.6% [9.4% (7.4%, 11.6%) and 65.2% (63.0%, 67.3%) for modes 2 and 4, respectively], and LVM with *R*^2^ = 61.7% [47.8% (45.0%, 50.6%) and 13.9% (11.5%, 16.4%) for modes 2 and 4, respectively]. MVR (and a portion of LVM) variability was captured by modes that explained less of the LV shape variability (modes 8 and 16–19), with the sum of coefficients of determination *R*^2^ = 79.63% (78.2%, 81.0%).

### Relationships between biometric parameters and LV shape modes

**Figure 4:**
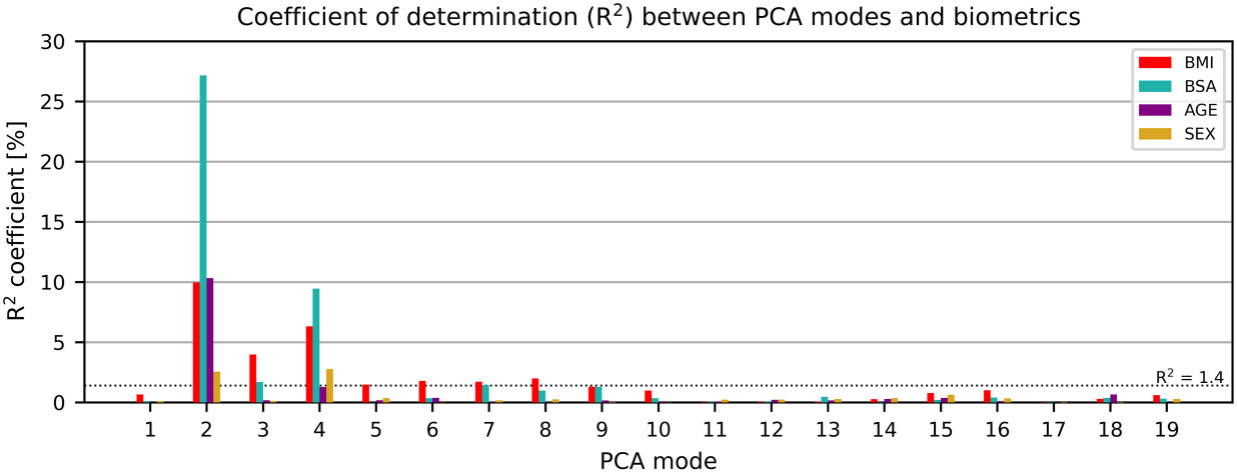
Correlation between the PCA modes and biometric parameters. Modes depicting length, sphericity, and wall thickness were highly correlated with body mass index (BMI), body surface area (BSA), and age. Modes 3, 5, 6, and 8 were mainly correlated with BMI, which signals that they may represent the morphological remodelling related to obesity. PCA, principal component analysis.

Of the biometric parameters, BSA had the strongest correlation with LV shape and was mostly associated with modes 2 and 4, *R*^2^ of 27.2% (24.3%, 30.1%) and 9.4% (7.4%, 11.7%), respectively (see *Figure 4*). Larger BSA corresponded to a larger and wider LV, with an increase in overall size and length almost three times as big as in sphericity, and to increased mass without changes in MVR.

Age was second most correlated with LV shape, mainly with mode 2 [*R*^2^ = 10.3% (8.2%, 12.6%)] and to a lesser extent with mode 4 [*R*^2^ = 1.3% (0.6%, 2.3%)]. The left ventricle, developing within this age range, increases in size, mass, and sphericity without changes in MVR.

Sex was the weakest correlated parameter with LV shape, almost equally correlated with mode 4 [*R*^2^ = 2.8% (1.7%, 4.1%)] as with mode 2 [*R*^2^ = 2.6% (1.5%, 3.9%)]. Boys have slightly but statistically significantly larger (average LVEDV of 100.0 ± 16.6 mL vs. 93.4 ± 16.9 mL, *P* < 0.001) and less spherical (average sphericity of 1.56 ± 0.14 vs. 1.60 ± 0.15, *P* < 0.001) left ventricles compared to girls.

### The LV anatomical signature uniquely associated to obesity and its onset

Similarly, BMI was mostly associated with size [mode 2, *R*^2^ = 10.0% (7.9%, 12.2%)] and sphericity [mode 4, *R*^2^ = 6.3% (4.6%, 8.2%)]. However, BMI was also associated with modes that were not or only weakly correlated with age, sex or BSA, namely modes 3, 5, 6 and 8 [*R*^2^ of 4.0%, 1.5%, 1.8%, and 2.0% respectively, with 95% confidence intervals of (2.6%, 5.6%), (0.7%, 2.5%), (0.9%, 2.9%), and (1.1%, 3.2%) respectively].

**Figure 5:**
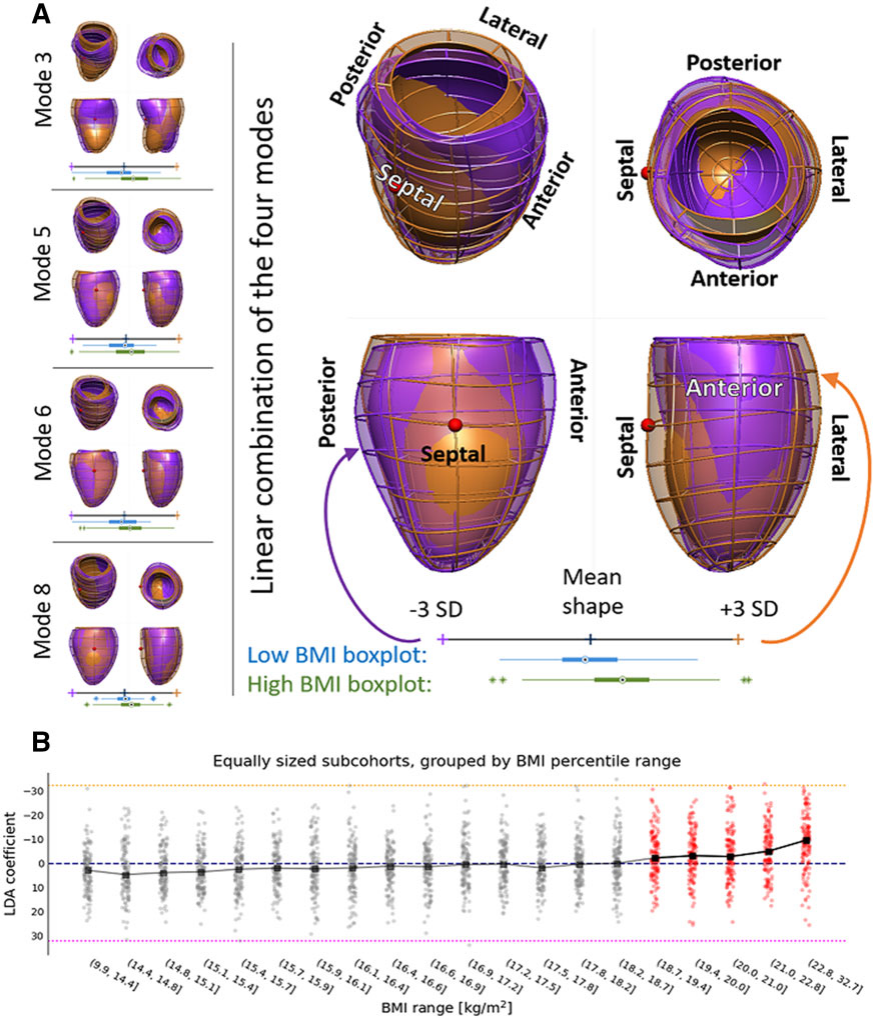
The signature of obesity in LV shape. (*A*) Individual modes (left) and their linear combination (right) that are specifically related to obesity. The shapes are shown from the septal and the anterior sides. The hearts of obese children (orange overlay) are more spherical, compressed in the antero-inferoseptal axis and have a more circular cross-section compared to children with low body mass index (BMI, purple overlay). The red sphere indicates the location of right ventricle. Distributions of high BMI (green) and low BMI (blue) young heart shape modes are provided in boxplots. PCA, principal component analysis; SD, standard deviation. (*B*) Scores of the signature of obesity in our cohort binned in ten equally sized samples. The inflection point is found within the 18.7–19.4 BMI (*P*-value of the *t*-test comparing previous and this bin equal to 0.006), suggesting that the young heart morphology becomes prone to remodelling around the BMI of 19.

The optimal linear combination of modes 3, 5, 6, and 8 that discriminated (linear discriminant analysis *P* < 0.001, receiver operating characteristic area under the curve: 0.71) between the 214 control cases (BMI = 17.5 ± 0.3 kg/m^2^) and the 185 overweight or obese cases (BMI > 22.8 kg/m^2^) in our cohort revealed the unique signature of obesity in the child’s heart as shown in *Figure 5*: with increasing BMI, the left ventricle had an increased end-diastolic volume especially in the apical and mid segments, as well as increased anteroseptal convexity. Moreover, in obese children, the basal cross-section became more circular, compared to the elliptical shape associated with lower BMI, while also compressing the left ventricle in the antero-posterior direction. The significance levels and power analysis of these results are depicted in Supplementary data online, *Table S5*. This signature of obesity did not have a linear relationship with BMI across the spectrum, but became apparent from a BMI of 19 kg/m^2^ onwards, see panel B in *Figure 5*.

## Discussion

This is the first study to characterize the paediatric heart in 3D, based on a comprehensive set of CMR scans of 2631 children. It may serve as a reference standard for future cohort studies or individual patient data. Moreover, we revealed that obesity in children is associated with unique morphological signatures, characterized by an increased apical and midventricular volume, anteroseptal convexity, anteroposterior compression, and rounding of the basal and mid segments. A web service hosted at the Cardiac Atlas Project (http://www.cardiacatlas.org, 12 December 2021, date last accessed) is released to enable an easy access to the novel morphological descriptors.

### Morphological variability of the paediatric LV

There is considerable data on the association of sex, growth, and body size with LVM and dimensions. Cross-sectional studies of healthy children, adolescents, and young adults found that measures of body size and adiposity are the strongest correlates, reflecting greater resting LV stroke volume and output.^24–27^ A paediatric statistical shape study could further assess and detect different phenotypes with potential prognostic information. Multiple statistical shape studies have been performed on large adult cohorts,^10,28^ yet the developmental path of the heart shape remains understudied. The provided description of paediatric LV shape variability fills one of the knowledge gaps on cardiac growth and development.^8^ This SSM has a potential to significantly aid the detection of individual and group changes in the healthy developmental patterns while considering various biometric parameters.

The 3D LV meshes were generated with sub-voxel accuracy and showed a good agreement in the metrics resulting from the contours. The mean difference between contour-derived and mesh-derived metrics was lower than the inter-observer variation between two experienced reviewers in a multicentre paediatric CMR study.^29^ Small discrepancies were caused by the impact of breath-hold artefacts on the 3D meshes, differences in how to account for uncomplete basal slices, and numerical differences between using the summation of disks and integration.

Variation in size, sphericity, and orientation of the LV were the main features captured by the first four modes of the paediatric LV, similarly to those observed in the adult populations.^10,28^ The associations of shape with age and BSA suggested that as a child gets older, the heart mainly increases in size and length, more than it increases in sphericity and wall thickness. Conversely, sex was equally correlated to changes in size and sphericity, with girls having smaller, more spherical hearts than boys, in-line with the adult population studies.^28^ Variation in MVR is encoded by modes 16–19, much smaller in the PCA ranking, and not related to BSA or BMI.

### Shape features related to obesity

Echocardiographic studies, supplemented by recent CMR investigations, have consistently indicated that LVM and dimensions are increased in the obese child.^13,30^ This association seems to be driven by the increased lean body mass accompanying obesity, not total body fat or blood pressure.^31^ Within the Generation R study, and based on traditional analyses of the same CMR data, childhood BMI positively associated with LV size, and this association was stronger for lean mass than for fat mass.^13^ Our SSM has shown a similar pattern: mode 2 (depicting size) had a 2.7-times stronger correlation with BSA (a measure more closely related to lean mass) than with BMI. BSA also had a 1.5-times stronger association with modes related to sphericity and wall thickness than BMI. The discovered shape features showed stronger unique association with obesity than the clinical LV measurements, including LVEDV, LVM, length, eccentricity, and others (see Supplementary data online, *Table S5*).

Shape atlases in adults found that sphericity was a key feature in predicting adverse outcomes.^32^ Sphericity is known to associate with maladaptive remodelling processes in asymptomatic populations and patients with obesity and angina,^32^ CVD,^33^ and increased adverse risk.^34^ In our study, LV sphericity was mainly captured in mode 4 and was significantly correlated with increased BMI. Nevertheless, it is unlikely that greater sphericity is purely maladaptive in the paediatric heart, given the strong correlation with BSA (see Supplementary data online, *Table S4*). Our analyses revealed a 3D anatomical remodelling pattern that is unique for obesity and cannot be expressed in traditional cardiac measures (*Figure 5*): children with a higher BMI had a decreased eccentricity of the LV cross-section and the LV apex tilted towards the right ventricle. The results suggest that it is the mid and apical region of the heart that starts the progression towards adverse trait of sphericity, together with a decrease of eccentricity. Moreover, the ‘obese cardiac phenotype’ found in the paediatric LV had strong similarities with the cardiac remodelling patterns found in the presence of high afterload, i.e. aortic stenosis where LV shape was characterized by increased LV apical tilting towards the right ventricle,^35^ as captured in mode 3 in our study.

The rate of increase in regional stress relative to pressure is greatest in the septum,^36^ and our results reveal that adaptation to changes in BMI and BSA in modes 2 and 4 are localized to septal thickening (see Supplementary data online, *Table S6* and *Figure S4*) that agree with this mechanistic hypothesis. All thickening patterns observed in the modes correlated with change in LVM showed a relatively similar spatial distribution that concentrates in the septal wall, with a helical pattern that was described as characteristic in hypertrophic cardiomyopathy.^37^ Concentric adaptations to altered loading conditions may preferentially develop in an asymmetric, regional pattern.^36^ In our study, these reveal as asymmetric anatomical remodelling responses in terms of curvatures and volume distributions (see *Figure 5*). These changes may be explained by the pericardial loading conditions, with impact on end-diastolic morphology due to changes in thoracic pressures.

In contrast to adult studies,^9^ cardiac shape associations with obesity were similar for boys and girls. The identified shape features related to obesity in this study may explain how childhood obesity progresses to adult CVD. For example, modes 5, 6, and 8 described different manifestations of more circular cross-section along the left ventricle in subjects with higher BMI (see Supplementary data online, *Figure S5*). This observation mimics findings in adults, where subjects with higher peripheral (but not abdominal) fat mass had eccentric LV remodelling and a higher cardiac output.^38^ This indicates a heart adaptation to increased BMI by increasing both roundness of the shape and septal wall thickness to cope with higher work demand. Complex cardiac remodelling patterns may result in obesity from different stimuli, including haemodynamic loading conditions, obesity-associated pro-inflammatory factors, glucotoxicity, lipotoxicity, and leptin-resistance.^39^ The unique shape associated with obesity may serve as a biomarker that could identify those at highest risk for later CVD, including obesity-associated heart failure (the ‘obesity cardiomyopathy’). It also shows that remodelling associated with increased BMI can be regional and asymmetric.

### Limitations

The spatial orientation of the CMR short-axis slices stack may introduce image acquisition and 3D mesh variability as it is defined by the basal plane of the heart. In our study, modes 1 and 3 were mostly affected by this limitation, as they related to LV orientation in two perpendicular axes. Nevertheless, discrepancies in choice of the basal plane were random and could therefore be treated as noise—a noise shown to not affect LVEDV and ejection fraction.^40^ Due to the exquisite detail captured by an SSM, other subtle differences in methodological steps, such as CMR acquisition parameters and segmentation style, can also have an impact in the generality of results, and as such they should be the scope of future studies.^41^ Additionally, we chose the *R*^2^_BMI_ = 1.4 as a threshold to describe shape modes with the highest independent correlation to BMI. Other modes with lower signatures (including modes 10, 15, and 16) could be explored in future work, as they showed significance to increase in BMI (*P* < 0.001).

## Conclusions

We have described the variability in 3D shape of the paediatric heart, and identified unique anatomical features related to childhood obesity that could aid in risk stratification. A web service is released to provide easy access to the new shape parameters.

## Supplementary data

Supplementary data are available at *European Heart Journal - Cardiovascular Imaging* online.

## Supplementary Material

jeab271_Supplementary_Data

## Data Availability

The data underlying this article are partially available in Cardiac Atlas Project, at https://www.cardiacatlas.org/. The data include the 3D average mesh, and the meshes representing the shape modes. The meshes built from the CMR images cannot be shared publicly for the privacy of the individuals participating in the study. No new CMR images were generated in support of this research.
