# Supplementary material for: A three-dimensional atlas of child’s cardiac anatomy and the unique morphological alterations associated with obesity
Source: Eur Heart J Cardiovasc Imaging. 2021 Dec 21;23(12):1645–53. doi: 10.1093/ehjci/jeab271 (PMC9671403; doi:10.1093/ehjci/jeab271)
Supplement: jeab271_Supplementary_Data [file jeab271_supplementary_data.docx]

**A 3D atlas of child's cardiac anatomy and the unique morphological alterations associated with obesity**

Supplemental material

Maciej Marciniak^1^*, Arend W. van Deutekom^2,3,4^*, Liza Toemen^2,3,5^, Adam J. Lewandowski^4^, Romy Gaillard^2,3^, Alistair A. Young^1^, Vincent W.V. Jaddoe^2,3,5^, Pablo Lamata^1^

1 School of Biomedical Engineering and Imaging Sciences, Kings’ College London, London, United Kingdom;

2 Generation R Study Group, Erasmus MC, University Medical Center, Rotterdam, the Netherlands;

3 Department of Pediatrics, Erasmus MC, University Medical Center, Rotterdam, the Netherlands;

4 Oxford Cardiovascular Clinical Research Facility, Division of Cardiovascular Medicine, Radcliffe Department of Medicine, University of Oxford, Oxford, United Kingdom;

5 Department of Epidemiology, Erasmus MC, University Medical Center, Rotterdam, the Netherlands.

* Joint first authorship

**Contents:**

**Supplementary Methods**

- Anthropometrics
- CMR Imaging protocol and image analysis
- Disentanglement of the first two modes

**Supplementary Results**

- Flowchart of study participants
- Baseline characteristics of study participants with overweight or obesity compared to those with normal weight
- Validity of the generated meshes
- Shape modes visualisation and explained variance
- Linear discriminant analysis on 19 modes
- Power analysis to investigate the association of SSM of pediatric LV with obesity and BMI
- Coefficients of determination for the linear relationships between the geometric measurements and biometric parameters
- Variation of the wall thickness in selected modes
- Variation of the wall thickness in the LDA axis related to increased BMI

**Supplementary Discussion**

- On modes 5 and 6

## **Supplementary Methods**

### **Anthropometrics**

Children’s height and weight was measured in light clothing without shoes. All measurements were performed by trained research staff. Height was measured to the nearest millimetre by a stadiometer (Holtain Limited, Crosswell, Crymych, UK). Weight was measured to the nearest gram using an electronic scale (SECA 888, Almere, The Netherlands). BMI and body surface area (BSA, using the Haycock formula(18)) were calculated. Sex- and age-specific BMI standard deviation scores were obtained based on national reference diagrams.(19) Children’s weight status was defined according to age- and sex-specific cut-off points proposed by the International Obesity Task Force.(20)

### **CMR imaging protocol and image analysis**

We performed CMR imaging using a 3T GE scanner. Children were introduced with the scanning environment through the use of a simulated scanning session. CMR scanning was performed on a wide-bore GE Discovery MR 750 3T scanner (General Electric, Milwaukee, MI, USA). We acquired localizer images, followed by 2-chamber and 4-chamber views with ECG gated breath-held scans lasting less than 10 seconds per breath-hold. A short-axis steady state free precession cine stack was then obtained with basal slice alignment and covering the ventricles and part of the atria with contiguous 8-mm thick slices over several end expiration breath-holds. The imaging parameters for the short axis acquisitions were as follow: field of view of 280 x 280 mm^2^, scan matrix of 128 x 128; 16 views per segment, repetition time 3.7 ms, echo time 1.7 ms, flip angle 45°. The 2-chamber, 4-chamber and the short axis SSFP scans were stored on a digital archive for post-processing.

Off-line image analyses for LV measures on the short-axis cine stack was outsourced to a commercial party and analysed by 4 analysts under supervision of an experienced radiologist (Precision Image Analysis, Kirkland, WA, USA), using Medis QMASS software (Medis, Leiden, the Netherlands). LV short-axis endocardial and LV epicardial borders were semi-automatically contoured at end-diastole and end-systole to allow automated calculation of right and left ventricular volume and LVM, following the guidelines of the Society for Cardiovascular Magnetic Resonance (SCMR). Papillary muscle was included in the ventricular cavity. LVM was calculated as (diastolic epicardial volume-diastolic endocardial volume)×1.05. Interobserver intraclass correlation coefficients (ICC) were calculated in a random subset of 25 scans. For LVED, the ICC was 0.93 (95% Confidence Interval (CI) 0.88-0.97). ICC for LVM was 0.80 (95% CI 0.68-0.90).

## **Disentanglement of the first two modes**

When analyzing the infant left ventricular shapes and building the statistical shape model, the two first original PCA modes showed a mixed-up effect of two factors: apical tilting and the overall size attributed mainly to length. These two modes showed a similar and large amount of variance explained (22.9% and 19%), indicating that their definition was somehow arbitrary and not really dictated by the data. We therefore rotated the axes of variation in this 2-dimensional space to find the axis that was capturing the size effect. Univariate partial least squares (PLS) was used to disentangle the first two PCA modes.

We used the PLS on the first two modes only, for two reasons. Firstly, PCA is an unsupervised method of feature extraction and allows for search of features that are not predetermined yet associated with the parameters of interest. Secondly, the smaller shape modes generated with PLS are correlated with the parameters beyond the applied target labels, due to non-linearity embedded in the distributions of shapes and interdependence of the clinical indices (1). It is therefore counterproductive to apply PLS to all modes; however, it is desirable for the interpretability of the results to obtain a single mode associated solely to volume.

End-diastolic volume was set as a target variable and the coefficients of the 2 modes served as the independent variables. With univariate PLS, linear combinations of the explanatory variables related to the target variable are formed sequentially by ordinary least squares regression. These linear components, may be viewed as weighted averages of predictors, where each predictor holds the residual information in an explanatory variable that is not contained in earlier components, and the quantity to be predicted is the vector of residuals from regressing the target variable against earlier components. The acquired weights (here called coefficients) were depicted as mode 1 and mode 2 without the loss of information in Figure S1.

PLS keeps the range of the PCA scores and improves the correlation between the mode and volume from 0.75 to 0.81. The first PLS mode captures all correlation of the two PCA modes to the volume. Since the new mode related to volume explains less variance (relative explained variance of PLS mode 1 is 22.54 % whereas PLS mode 2 explains 19.42 %, of variance), it is still denoted as mode 2.


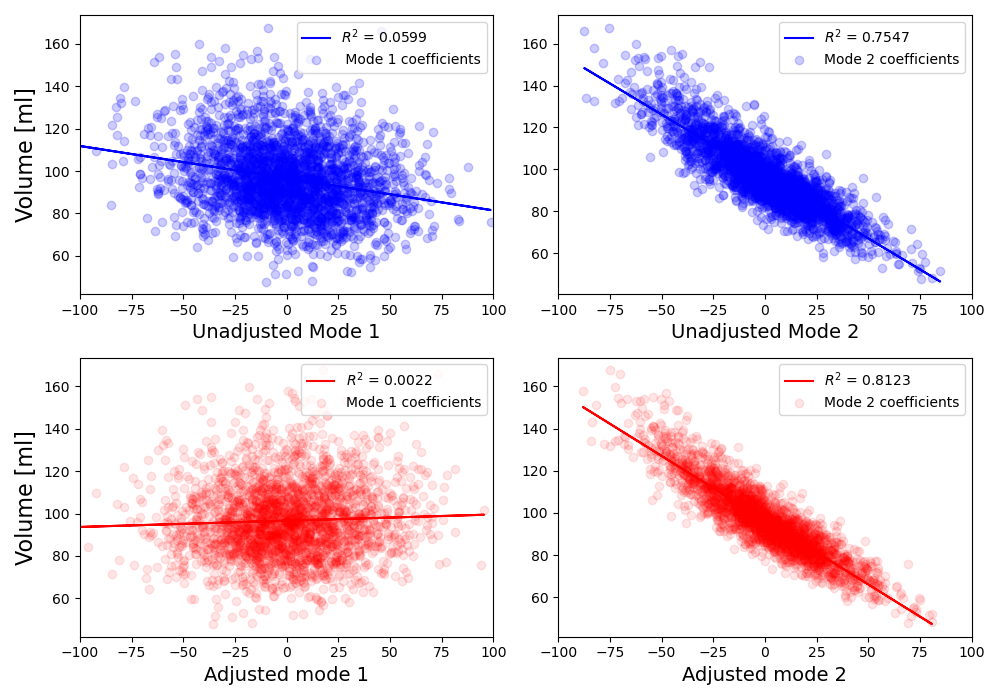


**Figure S1. Scatter plot of the original modes and modes adjusted with PLS in relation to the end-diastolic volumes.** The first adjusted mode (bottom-left, red) is not significantly correlated to the volume. At the same time, the second adjusted mode (bottom-right) is stronger correlated to volume than the 2 PCA modes (blue).

**Supplementary Results**


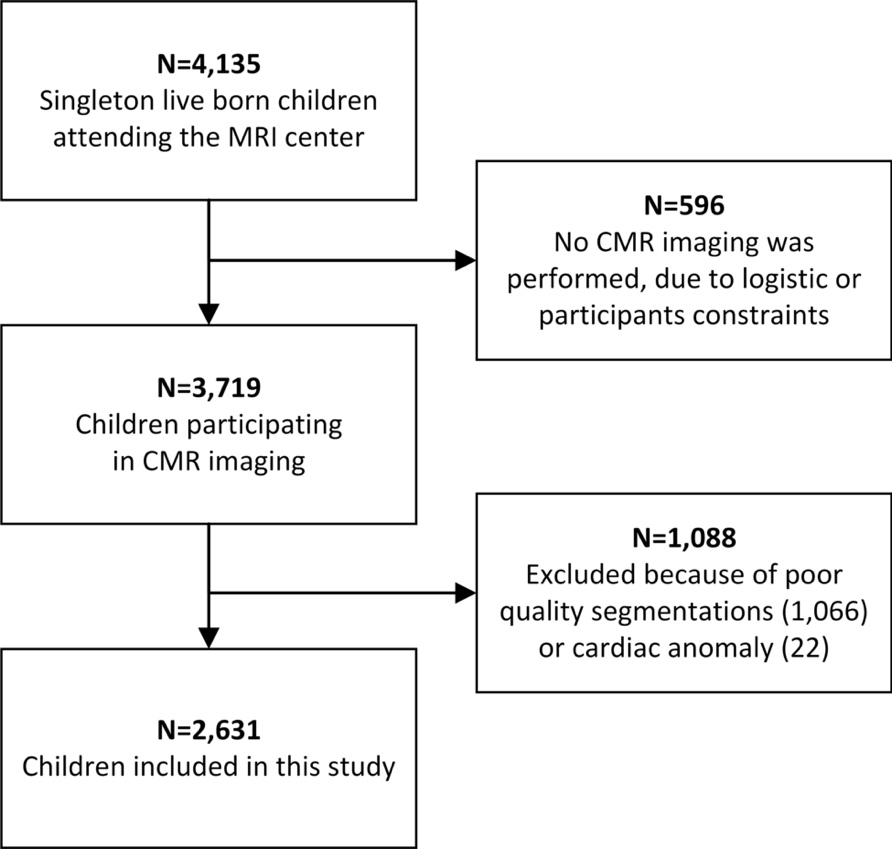


**Figure S2.** **Flowchart of study participants.** CMR images were considered low quality if more than 3 slices and/or the most basal or the most apical slices were missing from the cine short-axis stack, the quality of the images did not allow for segmentation or the breath-hold artefact distorted the position of the left ventricle such that the resulting mesh was not physiologically feasible. The segmentations were considered low quality if the number of segmented slices did not equal the number of short-axis slices, the endocardial or the epicardial contour of LV and/or endocardial contour of the right ventricle was missing.

**Table S1. Baseline characteristics of study participants with overweight or obesity compared to those with normal weight**

| **Variable** | **Overweight or obese subjects** | **Normal weight subjects*** |
| --- | --- | --- |
| Number of participants | 185 | 2,397 |
| Age at MRI, years | 10.3 (0.6) | 10.2 (0.6) |
| Male sex, n (%) | 74 (40.0%) | 1178 (49.1%) |
| Western European ethnicity, n (%) | 80 (44.7%) | 1682 (71.5%) |
| Height, cm | 145.2 (7.3) | 141.5 (6.4) |
| Weight, kg | 51.1 (7.2) | 34.3 (5.4) |
| Body mass index, kg/m^2^ | 24.1 (2.1) | 17.1 (1.9) |
| Body surface area, m^2^ | 1.44 (0.13) | 1.15 (0.11) |
| Left ventricular end-diastolic volume, ml | 114.2 (19.3) | 99.9 (16.8) |
| Left ventricular mass, g | 57.2 (10.3) | 48.4 (9.9) |

* Excluding 49 underweight subjects.

All differences between overweight or obese and normal weight children are statistically significant, due to the large study population. MRI indicates magnetic resonance imaging; SD, standard deviation.

### **Validity of the generated models**

The mesh personalization process achieved sub-voxel accuracy, with the average distance between initial contours and mesh surfaces of 0.7±0.3mm. LVEDV and LVM derived from the computational models agreed well with LVEDV and LVM derived from manual contours.


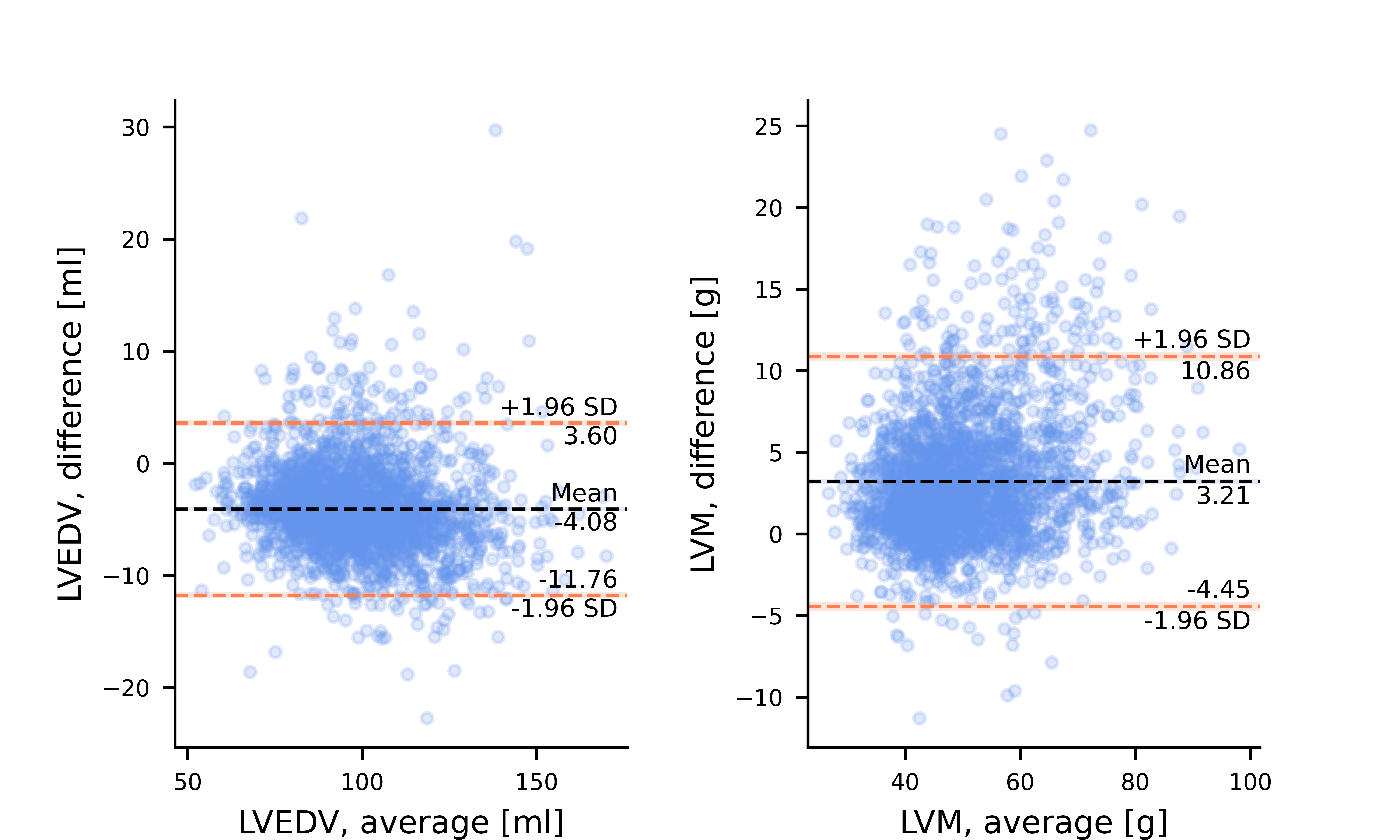


**Figure S3: Bland-Altman agreement plots between our cardiac meshes and manual contours**. Left: left ventricular end-diastolic volume (LVEDV) was on average underestimated by 4.1±3.8 ml, Right: left ventricular mass (LVM) was on average overestimated by 3.2±3.9 g.

## **Shape modes visualisation and explained variance**

**Table S2. 19 modes of shape.**

| Mode | Shape variation | Mode | Shape variation |
| --- | --- | --- | --- |
| 1 | 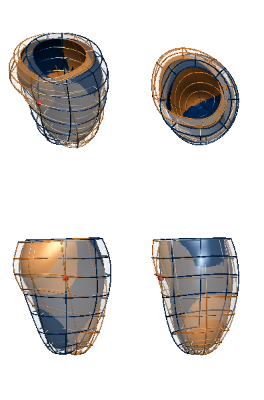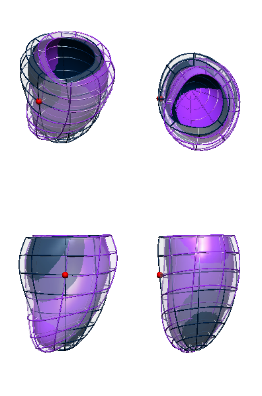 | 2 | 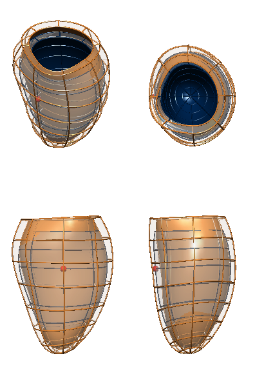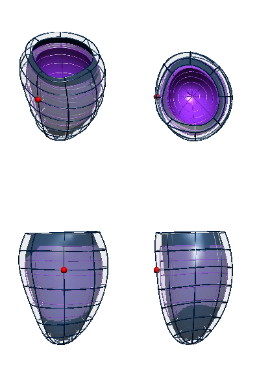 |
| 3 | 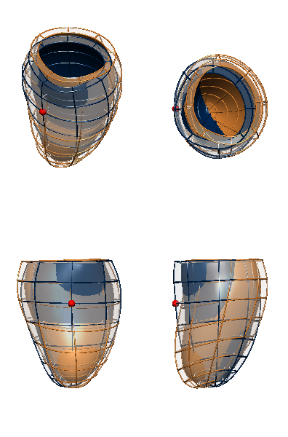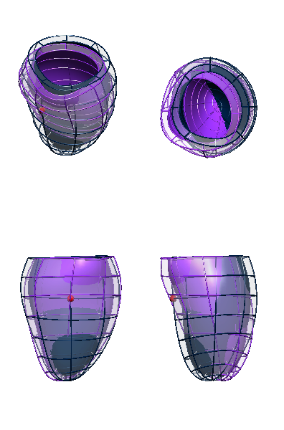 | 4 | 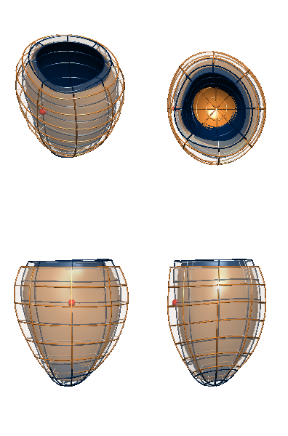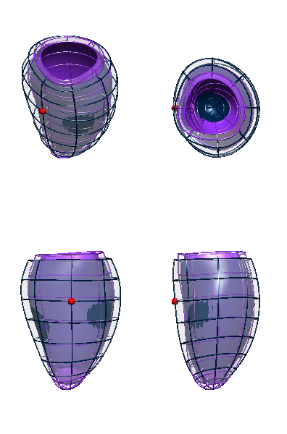 |
| 5 | 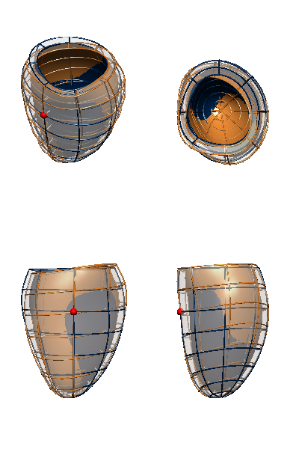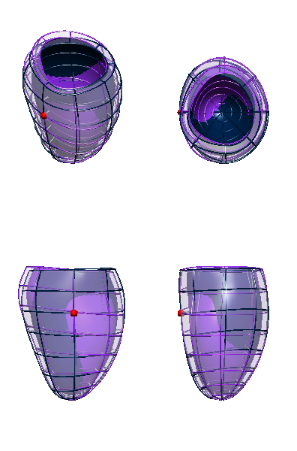 | 6 | 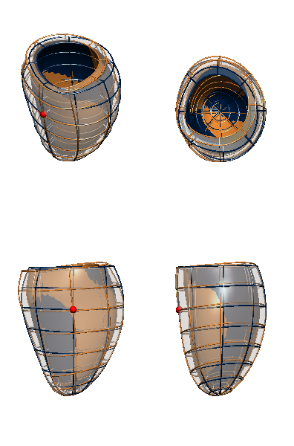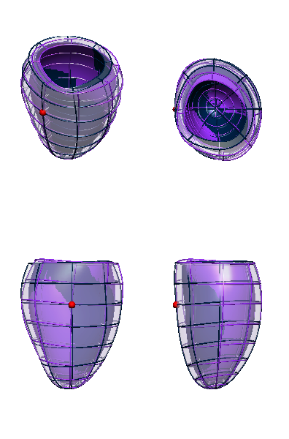 |
| 7 | 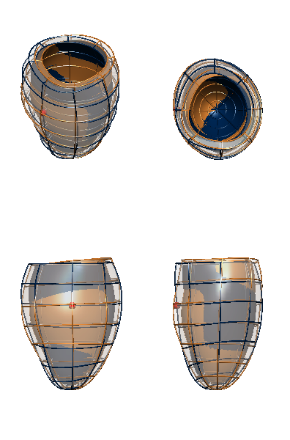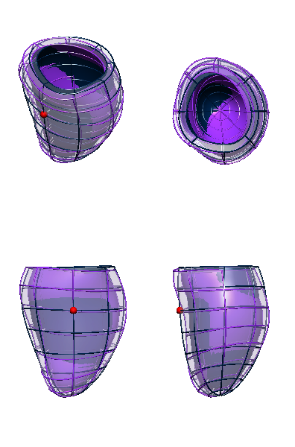 | 8 | 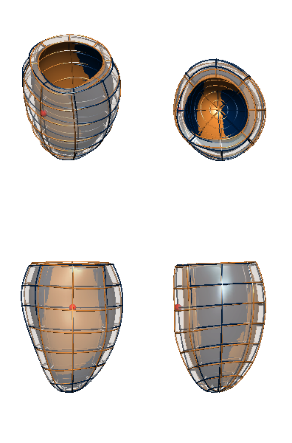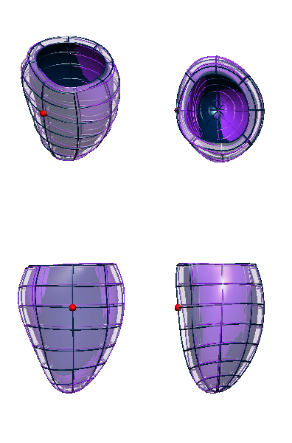 |
| 9 | 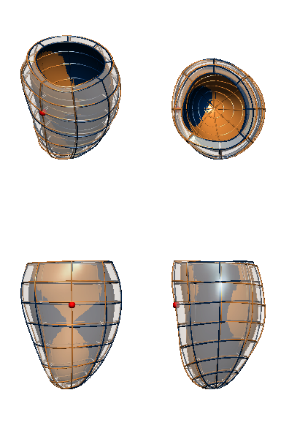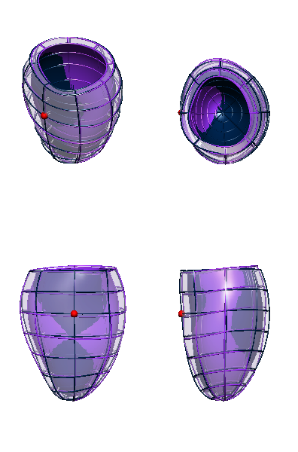 | 10 | 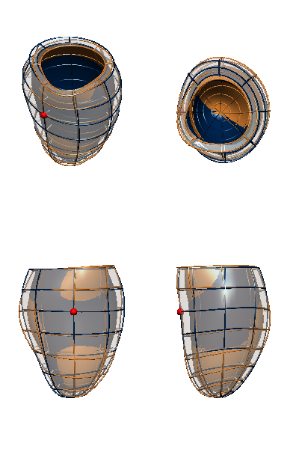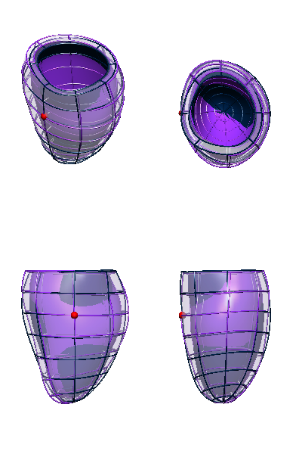 |
| 11 | 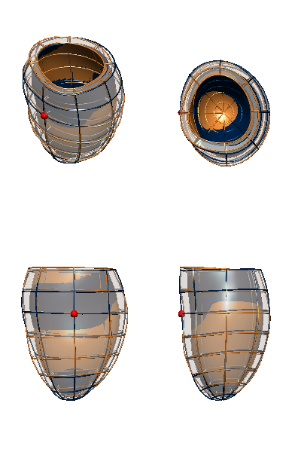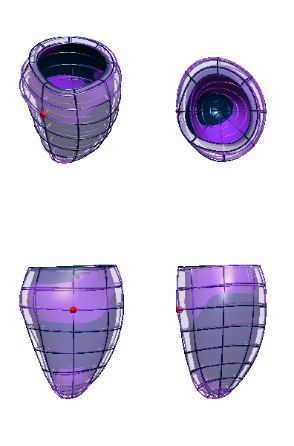 | 12 | 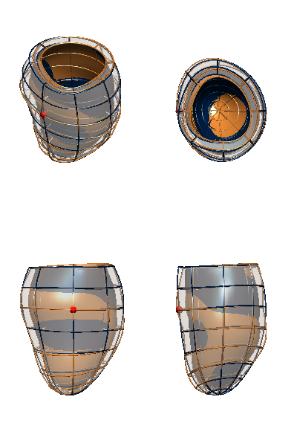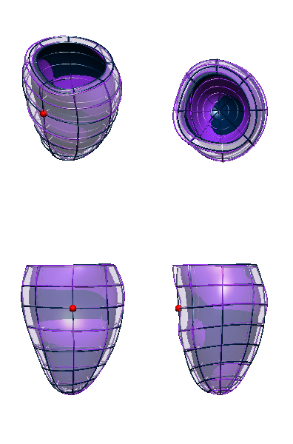 |
| 13 | 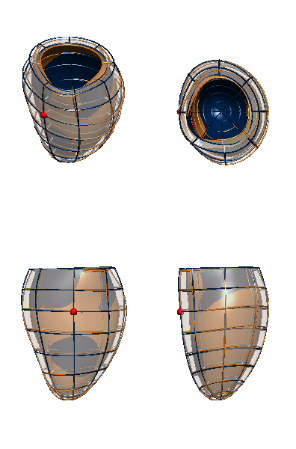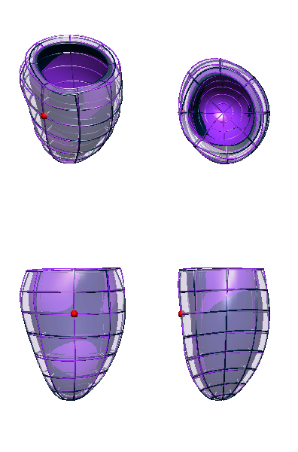 | 14 | 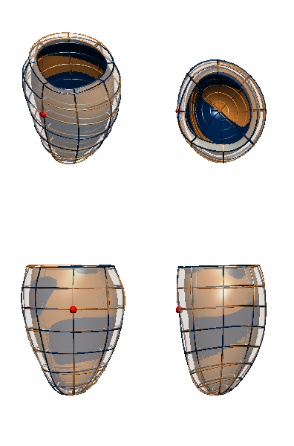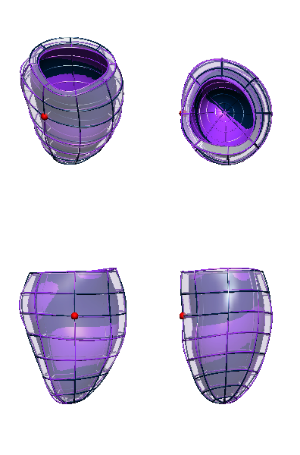 |
| 15 | 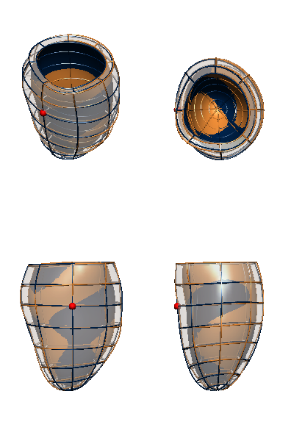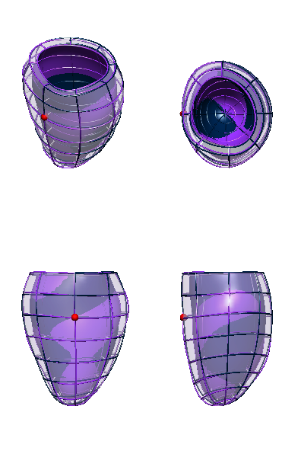 | 16 | 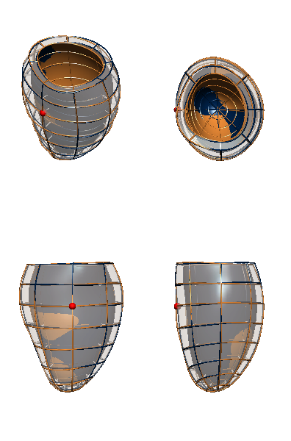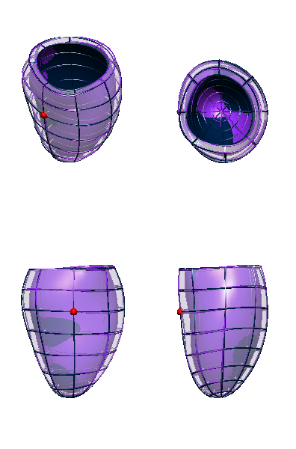 |
| 17 | 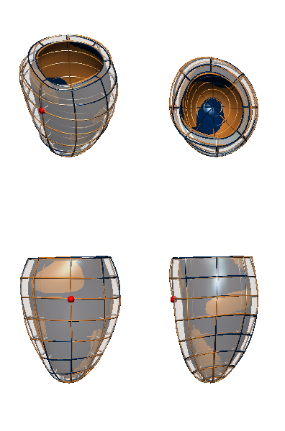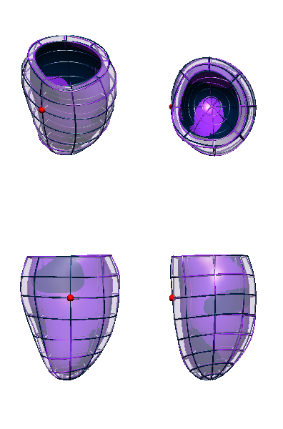 | 18 | 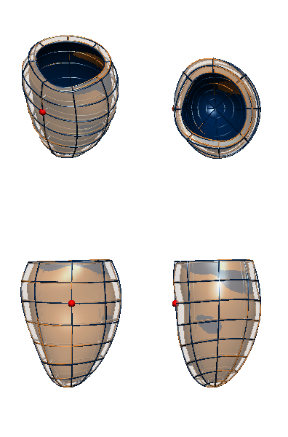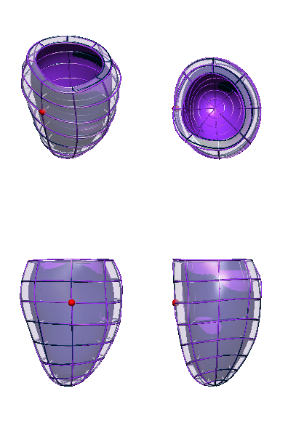 |
| 19 | 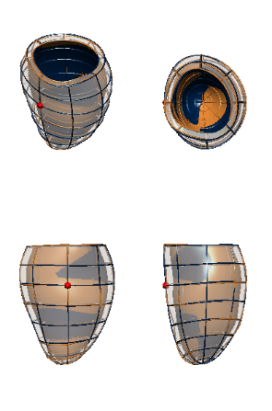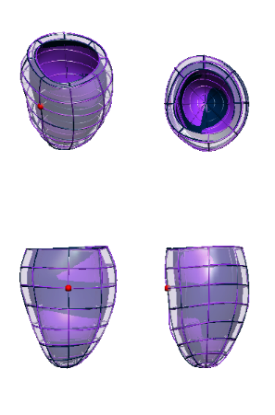 | Mean shape  +3 standard deviations along the mode  -3 standard deviations along the mode | |

**Table S3. Variance explained by modes.**

| **Mode** | **Relative explained variance (%)** | **Cumulative explained variance (%)** |
| --- | --- | --- |
| 1 | 22.54 | 22.54 |
| 2 | 19.42 | 41.96 |
| 3 | 13.96 | 55.93 |
| 4 | 11.18 | 67.10 |
| 5 | 4.44 | 71.54 |
| 6 | 3.39 | 74.92 |
| 7 | 2.96 | 77.89 |
| 8 | 2.36 | **80.25** |
| 9 | 2.07 | 82.31 |
| 10 | 1.88 | 84.20 |
| 11 | 1.44 | 85.63 |
| 12 | 0.96 | 86.59 |
| 13 | 0.77 | 87.36 |
| 14 | 0.70 | 88.05 |
| 15 | 0.61 | 88.66 |
| 16 | 0.54 | 89.20 |
| 17 | 0.50 | 89.70 |
| 18 | 0.47 | **90.17** |
| 19 | 0.43 | 90.59 |

## **Linear discriminant analysis on 19 modes**

The increase in body surface area (BSA) and body mass index (BMI) impacts the remodelling patterns of the left ventricle. The main changes are in size (volume in mode 2 and diameter/sphericity in mode 4, see Figure 5 in the main manuscript). The goal was to find the single linear anatomical change (mode) that best explained changes in BSA and BMI. We extracted this single linear direction of remodelling with the linear discriminant analysis (LDA).

In more detail, we considered the first 19 modes, which were related to size, sphericity, wall thickness and length. In order to see the differences between the two patterns, we adjusted the PCA coefficients for BMI and BSA using the PLS regression method described above. We then performed the LDA on all relevant cases, as described in the main text. The results of the LDA for unadjusted and adjusted coefficients are presented in Table S4.

Visual inspection revealed that these directions are similar to one another as it was expected, since both high BMI and BSA cause an increase in the afterload reflected with size and dilation (especially in the septo-lateral direction, increasing sphericity) as key anatomical features. The result suggests that a single linear direction is too much of a simplification for the comprehensive description of the remodelling pattern. However, the 4 modes used to identify the BMI specific directions of anatomical change, that were found using the criteria based on the correlation coefficient are more strongly correlated with the BMI than BSA.

**Table S4. Linear discriminant analysis results on the set of all relevant modes.**

| Body Mass Index analysis with LDA | 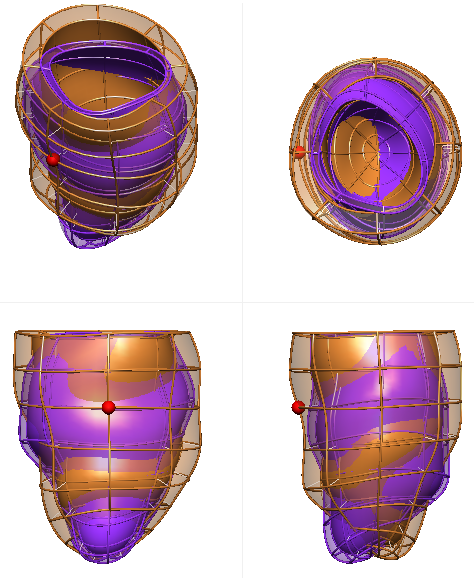 | 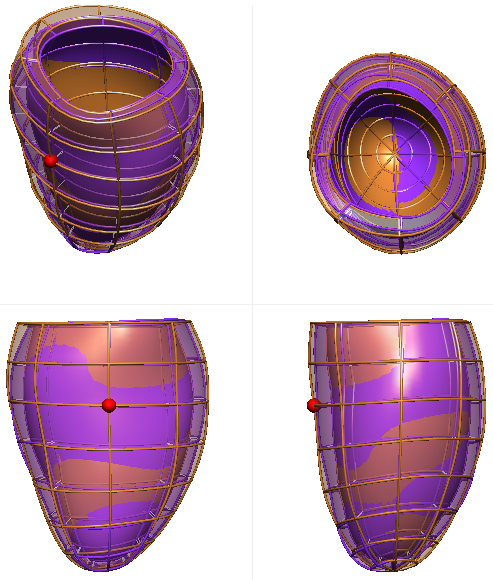 |
| --- | --- | --- |
| Body Surface Area analysis with LDA | 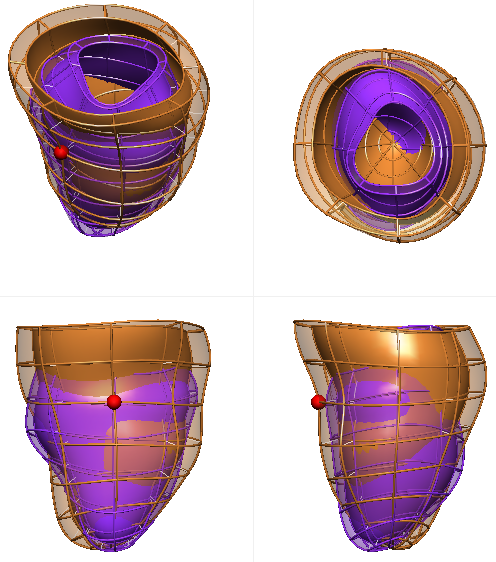 | 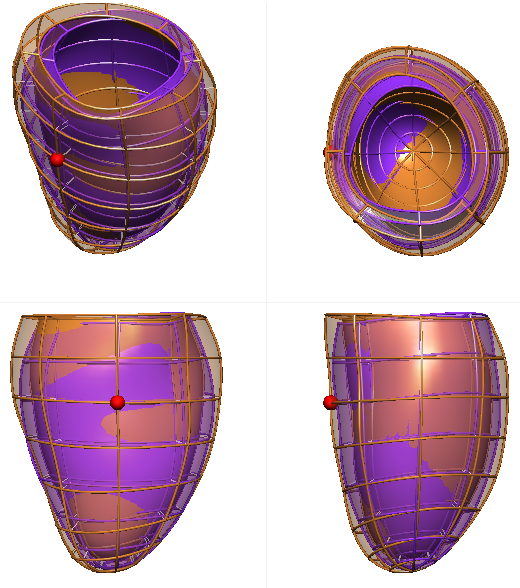 |
| Coefficients | Unadjusted | Adjusted |

Each panel presents the extremes of the linear anatomical change, with the orange extreme corresponding to the high BMI or BSA extreme, and purple to low BMI or BSA extreme.

**Power analysis to investigate the association of SSM of pediatric LV with obesity and BMI**

To calculate the sample sizes needed to detect a relevant simple correlation with specified significance level and power, we used the null hypothesis $H_{0}:\rho=0$ versus the alternative hypothesis $H_{a}:\rho\neq0$, where $\rho$ is the true correlation value. To calculate the sample size necessary to achieve the specified significance level and power, we calculated the Fisher’s arctanh transformation:

$$C\left( r \right)= \frac{1}{2}ln\left( \frac{1+r}{1-r} \right)$$

Given the sample correlation *r* based on *N* observations, which is distributed about $\rho$, $C\left( r \right)$ is normally distributed with the mean $C\left( \rho\right)$ and variance $\sigma^{2}= \frac{1}{N-3}$. The test statistics under the null hypothesis is

$$Z=C\left( r \right)\sqrt{N-3},$$

where $Z\sim N(0,1)$.

With this, the relevant sample size *N* is equal to

$$N= \left( \frac{Z_{\alpha}+Z_{\beta}}{C\left( r \right)} \right)^{2}+3,$$

where $Z_{\varphi}$ is the upper *100 (1-p)* percentile of the normal distribution, $\alpha$ corresponds to the probability of type I error, and therefore rejecting the true null hypothesis, whereas $\beta$ describes the probability of type II error, or not rejecting the false null hypothesis.

The correlation between the LDA coefficients found by combining modes 3, 5, 6 and 8 and the BMI was equal to 29.2%. Sample sizes were equal to $N_{1}=185$ and $N_{2}=214$. The maximum power of 95% was could have been achieved at the statistical significance level of 0.05. With smaller $\alpha$ of 0.01, the power of 90% could have been achieved. Finally, with $\alpha$ of 0.001, power of 75% could have been achieved. Table S5 shows the sample sizes necessary to achieve the desired statistical significance and power, given different values of $\alpha$and $\beta$.

**Table S5. Sample sizes necessary to achieve the statistical significance level and power, given the correlation between the shape coefficients and BMI.**

| $\boldsymbol{\alpha/1-\beta}$ | **75%** | **80%** | **90%** | **95%** |
| --- | --- | --- | --- | --- |
| **0.05** | 80 | 90 | 120 | 148 |
| **0.01** | 121 | 133 | 169 | 201 |
| **0.001** | 178 | 193 | 236 | 274 |

Cells marked with green color correspond to the achievable significance levels and power with the sample sizes used in the study. Cells marked with yellow color depict the sizes where only a single sample size was large enough to achieve the desired significance levels and power. Finally, cells marked with red color show that with the sample sizes used, the statistical significance or power cannot be achieved.

**Table S6. Coefficients of determination for the linear relationships between the geometric measurements and biometric parameters.**

| **Coefficients of determination [%]** | **Body mass index** | **Body surface area** | **Age** | **Sex** |
| --- | --- | --- | --- | --- |
| End diastolic volume | **13.61** | **32.68** | **10.76** | **3.69** |
| Mass | **16.28** | **30.46** | **10.9** | **5.58** |
| Mass to volume ratio | **1.67** | **1.03** | **0.59** | **1.06** |
| Sphericity | **0.76** | **0.45** | 0 | **1.82** |
| Length | **4.71** | **14.68** | **6.74** | **0.81** |
| Average thickness | **8.19** | **11.19** | **3.97** | **3.27** |
| Conicity | 0.04 | 0.03 | 0 | **0.49** |
| Endocardial diameter at base | **12.67** | **27.92** | **7.29** | **3.31** |
| Endocardial diameter at mid | **8.67** | **20.11** | **5.79** | **4.91** |
| Endocardial diameter at apex | **4.14** | **9.91** | **2.71** | **3.09** |
| Endocardial eccentricity at base | 0.03 | 0.03 | 0.01 | 0 |
| Endocardial eccentricity at mid | **0.9** | 0.25 | 0.01 | 0.08 |
| Endocardial eccentricity at apex | 0.16 | 0.04 | 0 | 0.03 |

All geometric measurements were performed on the left ventricular models. The acquisition of the measurements is described in the main text. Only the endocardial eccentricity measured in the mid segments of the ventricle is correlated mainly to the body mass index with R^2^=0.9%, and no other parameters. Values shown as bold were statistically significant (p<0.001). Further description of the clinical measurement variability was described in (2).

**Table S7. Variation of the wall thickness in selected modes.**

| Mode | Thickness variation | Mode | Thickness variation |
| --- | --- | --- | --- |
| 2 | 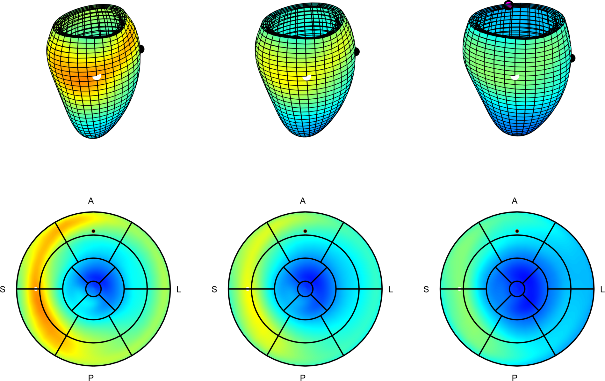 | 4 | 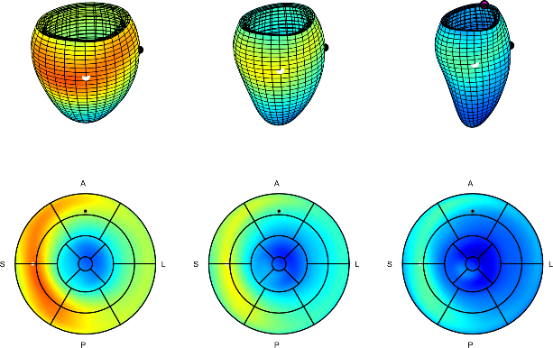 |
| 8 | 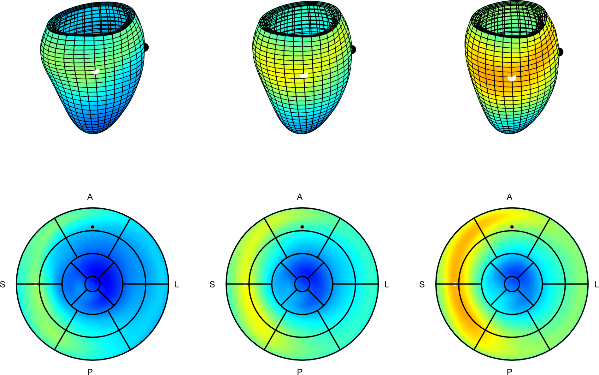 | 16 | 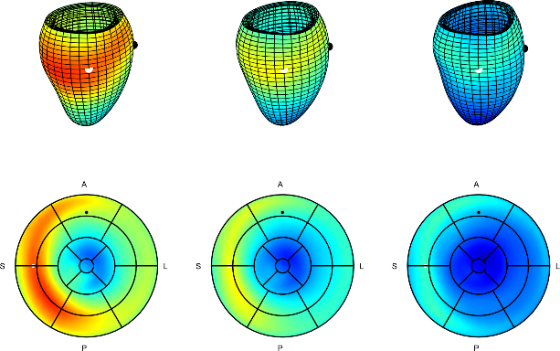 |
| 17 | 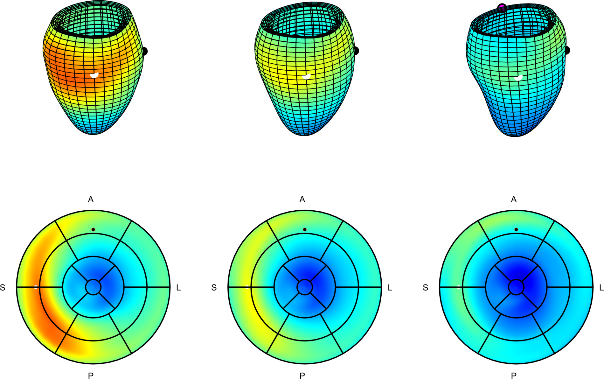 | 18 | 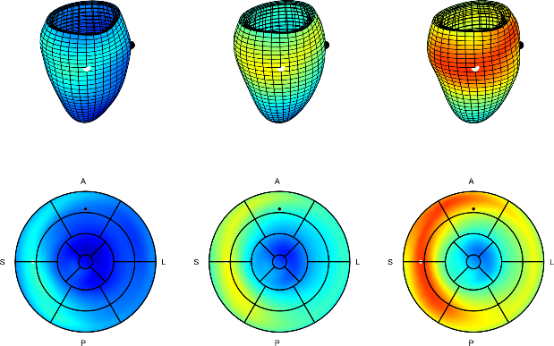 |
| 19 | 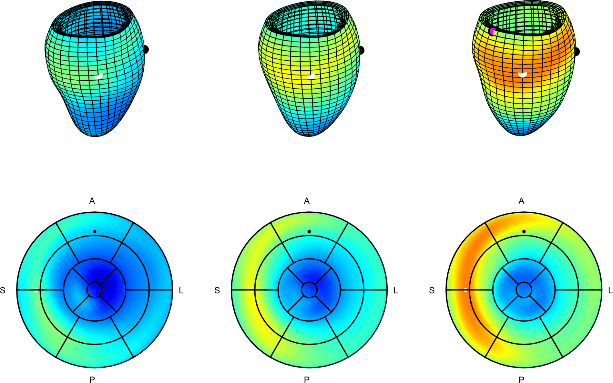 |  | Thickness [mm] 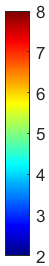 |

The visualisation of the wall thickness differences along the modes with coefficient of determination between the mode and left ventricular mass higher than 2%. Each figure presents minus (left) and plus (right) 3 standard deviation from the mean shape (middle). White and black spheres help to orient the plots, by pointing towards the middle of the septum and black towards the anterior of the LV. A stands for anterior; P, posterior; S, septal; L, lateral.

##
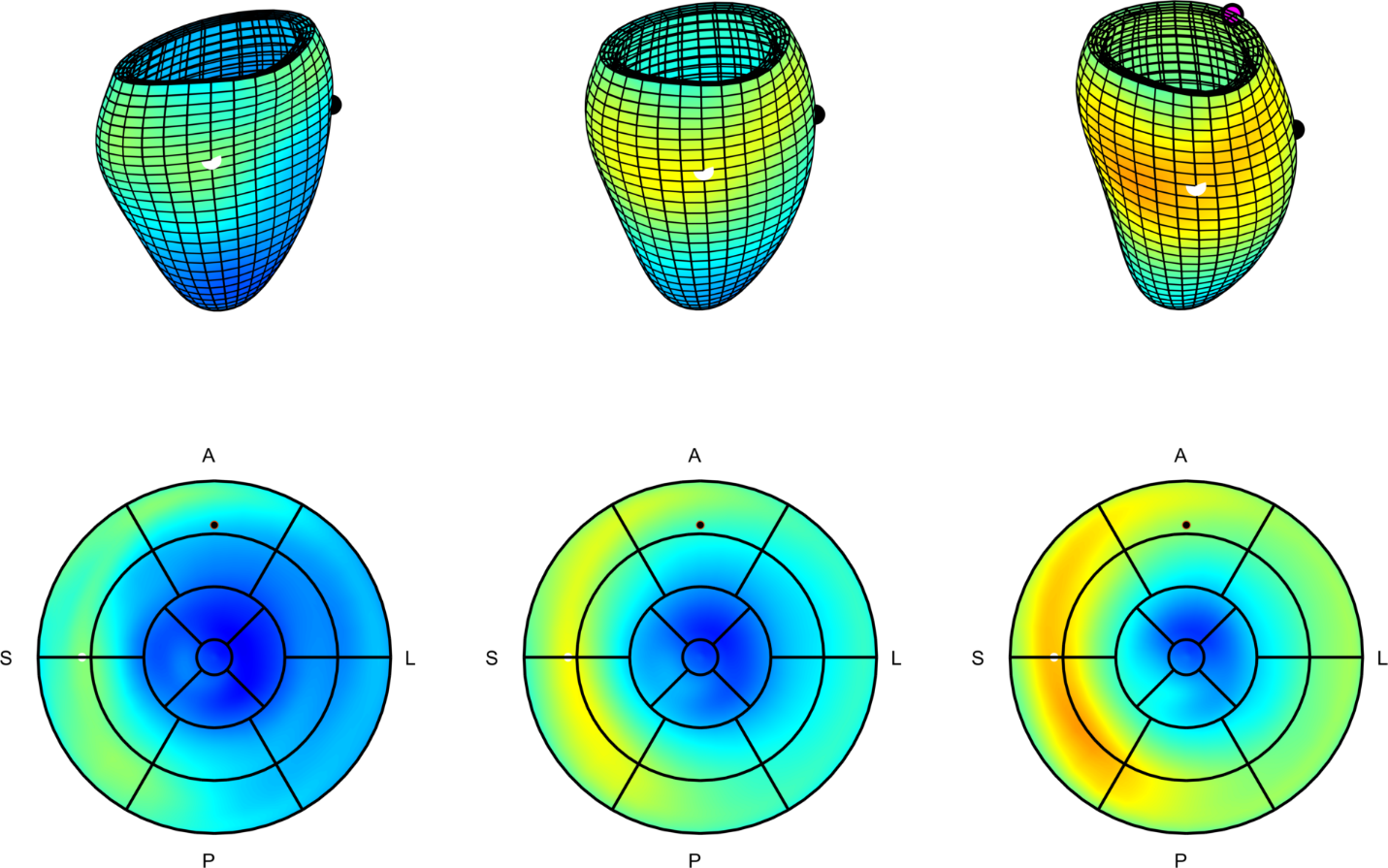


**Figure S4. Variation of the wall thickness in the LDA axis related to increased BMI.** The meshes and 17 AHA plots present minus (left) and plus (right) 3 standard deviation from the mean shape (middle) along the LDA axis. The differences in thickness result from differences in mode 8 and manifest with overall thickening of the LV in obese and overweight cases, most apparent in the base of posterior septum. On the other hand, decreased BMI manifests with thinner LV, with significant thinning in the basal anteroseptal segment, at the beginning of the LVOT. White and black spheres help to orient the 17 AHA plots, with point pointing towards the middle of the septum and black towards the anterior of the LV. A stands for anterior; P, posterior; S, septal; L, lateral.

## **Supplementary Discussion**

## **On modes 5 and 6**

Modes 5 and 6 correspond to LV changes its orientation w.r.t. RV, caused by compressing the LV in the antero-posterior direction (see Figure 2 in the main manuscript). These modes are present due to the methodology of the orientation registration conducted before the atlas construction. LV is oriented such that the axis between the centres of masses of the LV and RV are parallel and anchored in the centre of the LV for all models. This alignment allows for a potential source of variability related to the length of the RV in anteroposterior direction. The outlet (leading to pulmonary valve) component maybe elongate towards the anterior, without the change of the centre of mass, thus changing the orientation of the registration axis, visualised as a subtle rotation, which can be noticed by looking at the outflow tract (see Figure S5).


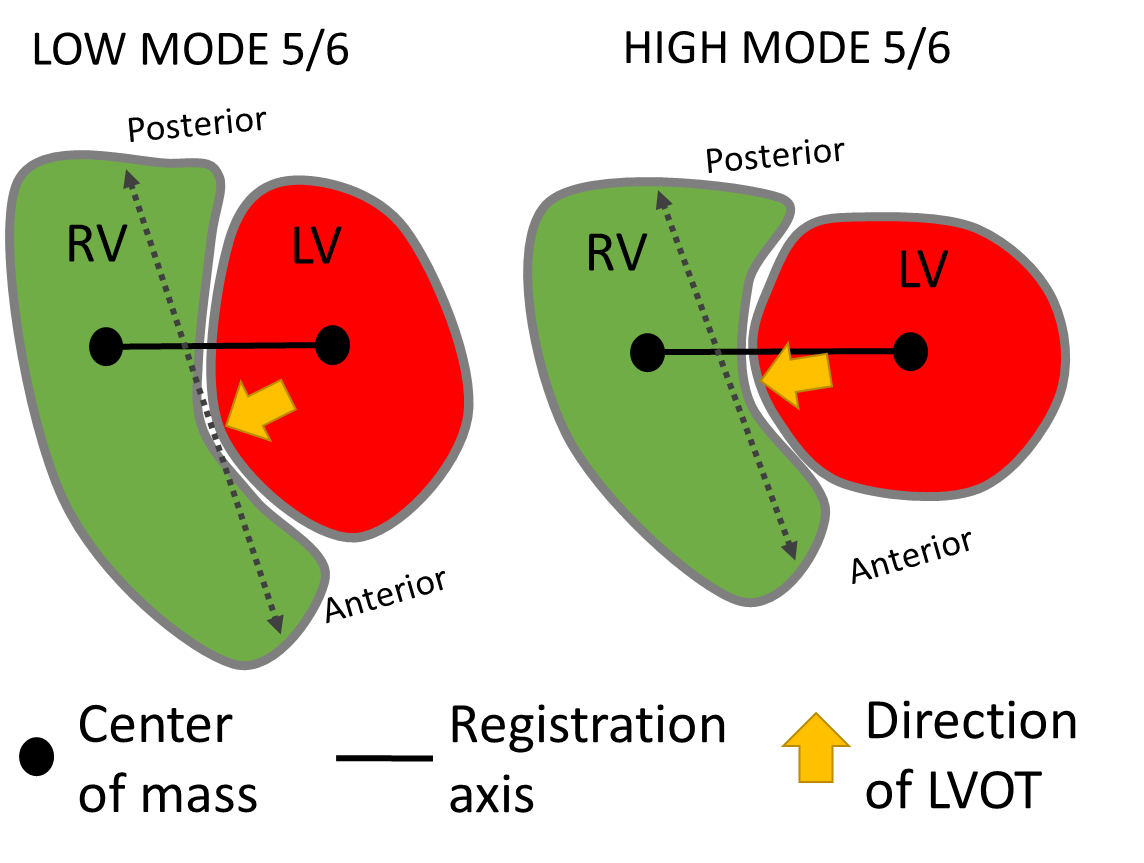


**Figure S5. Registration axis governing the orientation may be affected by the compression of the heart in anteroposterior direction.**

**Supplementary references**

1. Zhang X, Medrano-Gracia P, Ambale-Venkatesh B, et al. Orthogonal decomposition of left ventricular remodeling in myocardial infarction. Gigascience. 2017 Mar 1;6(3):1.

2. Toemen L, Santos S, Roest AA, et al. Body fat distribution, overweight, and cardiac structures in school-age children: A population-based cardiac magnetic resonance imaging study. J Am Heart Assoc. 2020 Jul 7;9(13):e014933.
